# Ionic liquids modified graphene oxide composites: a high efficient adsorbent for phthalates from aqueous solution

**DOI:** 10.1038/srep38417

**Published:** 2016-12-02

**Authors:** Xinguang Zhou, Yinglu Zhang, Zuteng Huang, Dingkun Lu, Anwei Zhu, Guoyue Shi

**Affiliations:** 1 School of Chemistry and Molecular Engineering, East China Normal University, 500 Dongchuan Road, Shanghai 200241, P. R. China

## Abstract

In 2015, more than 30% of erasers were found to contain a PAE content that exceeded the 0.1% limit established by the Quality and Technology Supervision Bureau of Jiangsu Province in China. Thus, strengthening the supervision and regulation of the PAE content in foods and supplies, in particular, remains necessary. Graphene oxide (GO) and its composites have drawn great interests as promising adsorbents for polar and nonpolar compounds. However, GO-based adsorbents are typically restricted by the difficult separation after treatment because of the high pressure in filtration and low density in centrifugation. Herein, a series of novel ionic liquids modified graphene oxide composites (GO-ILs) were prepared as adsorbents for phthalates (PAEs) in eraser samples, which overcame the conventional drawbacks. These novel composites have a combination of the high surface area of graphene oxide and the tunability of the ionic liquids. It is expected that the GO-ILs composites can be used as efficient adsorbents for PAEs from aqueous solution. This work also demonstrated a new technique for GO-based materials applied in sample preparation.

Phthalates (PAEs), which are used widely as plasticizers, elicit similar functionality to estrogen hormones in humans. For this reason, phthalates have adverse influences on the endocrine system and kill sperm. Therefore, PAEs have long attracted attention, especially since the so-called white spirit issue in China in 2012, in which significant phthalate contamination was uncovered. Subsequently, the PAEs content has been strictly monitored, especially in foods and supplies. However, in 2015, more than 30% of erasers were found to contain the PAEs content that exceeded the 0.1% limit established by the Quality and Technology Supervision Bureau of Jiangsu Province in China. Thus, strengthening the supervision and regulation of the PAE content in foods and supplies, in particular, remains necessary. Sensitive and accurate analytical detection of PAEs are in growing demand to address the problems of low concentration and complex matrix interferences from the environment and food sample^1^,^2^.

Graphene (G) has been widely studied in many research fields because of its superior physical and chemical properties. Structurally, graphene is a layered two-dimensional sheet bound by van der Waals forces^3^,^4^. This novel carbon material possesses an ultrahigh specific surface area^5^, high mechanical strength^6^ and excellent extraction capacity, making it a promising adsorbent^7^,^8^. Considering its large delocalized π-electron system, graphene can serve as good adsorbents for the extraction of hydrophobic and non-polar analytes^9^,^10^,^11^,^12^. In contrast, graphene oxide (GO), which can be prepared by the Hummers method, possesses abundant hydrophilic groups such as -OH and –COOH^13^ that can enhance the ability of GO to disperse in aqueous phase and form interfacial interactions with analytes. Based on these advantages, several groups have reported the application of GO as high efficient adsorbents^14^,^15^,^16^,^17^,^18^,^19^. However, the practical application of G or GO as adsorbents for solid phase extraction (SPE) have been limited because of problems associated with high column pressures, irreversible aggregation and their difficulty to recover from the liquid phase^20^. Thus, finding effective solutions for these challenges remains an issue.

GO-based composites have recently attracted intense interest in SPE. Many works have reported the preparation of GO-based magnetic composites as magnetic solid phase extraction (MSPE) adsorbents^21^,^22^,^23^,^24^. The magnetic adsorbent is dispersed in the sample solution and separated by an external magnetic field. This technique has been demonstrated to be effective in pre-concentrating inorganic and organic compounds from food, biological and environmental samples. However, in contrast to the standard fixed-bed column SPE techniques, the absorbents of MSPE must be magnetic and can be unstable^25^, which restricts its material scope and increases the difficulty of its preparation. Zhang *et al*.^26^ and Xu *et al*.^27^ reported the preparation of GO chemical-bonded to a solid phase microextraction (SPME) fiber via the reaction between –COOH in GO and a modified –NH_2_ on the fiber. Notably, the chemical bonding method improved the stability and reproducibility of the SPME fiber. Based on the fiber, the analytes can be extracted relatively quickly from complex media. However, the adsorption capacity of the fiber is limited. Liu *et al*.^20^ and Shi *et al*.^28^ prepared new SPE adsorbents by reduced graphene oxide (RGO) and GO sheets modified on silica. These composites not only preserve the advantages of GO but also overcome the problem of high column pressure during perfusion. For these reasons, novel GO-based composites applied as SPE adsorbents are promising materials and warrant further attention.

Ionic liquids (ILs) as a “designer liquid” have attracted significant attention because of their noteworthy and useful physicochemical properties, such as negligible vapor pressure, high thermal stability and high polarities^29^,^30^. In addition, ILs can combine with different functional materials to give superior properties by way of surface modifications, which extends the range of applications in many areas of chemistry^31^,^32^. IL-modified GO composites have been reported for use in sensitive electrochemical sensing and SPE^33^,^35^. For instance, Valentini *et al*. characterized GO/ILs-based screen printed electrodes^36^. The modified electrodes displayed an improved electrochemical performance, including increased peak current and electron-transfer efficiency. Ding *et al*. synthesized a magnetic chitosan and GO-IL composite material for the SPE of protein^37^. With the increased water solubility and interfacial interactions, the extraction efficiency of the proteins was enhanced. Therefore, with its unique properties, GO-ILs will give rise to more opportunities in material science and analysis chemistry.

In our work, we have prepared four types of amine-functionalized IL-modified GO composites that can be easily loaded into a syringe filter with a low back-pressure, which avoids damage to the SPE column while retaining the high adsorption capacity of GO (Fig. 1). With the analyses performed using a liquid chromatography-UV detector, the composites were used to extract nine PAEs from eraser samples, such as di(2-ethylhexyl) phthalate (DEHP), dibutyl phthalate (DBP), diisobutyl phthalate (DIBP) and so on, which are regarded as high-priority pollutants giving rise to breast cancer and lesion in male sex organ. Additionally, in order to optimize the method, the factors influencing the process of adsorption and desorption, such as pH, salt content and washing time were investigated. According to the data, the GO-IL composites not only possessed high adsorption capacity but also have good reproducibility. To the best of our knowledge, this is the first report where GO-IL composites have been packed into a column/cartridge for SPE. This approach provides a simple and effective solution to apply GO-based materials for adsorption.

## Results and Discussion

### Characterization of the GO-IL Composites

**Figure 2:**
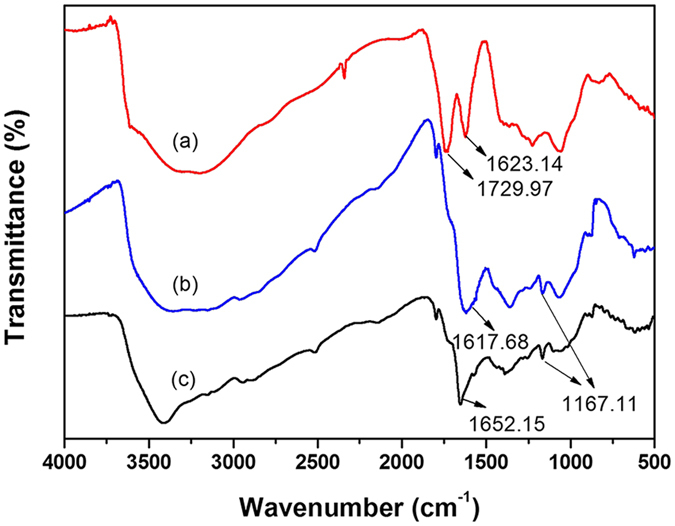
FTIR spectra of (**a**) GO, (**b**) GO-[AEMIM][Br], and (**c**) GO-[APMIM][NTf(2)].

Figure 2 shows the FTIR spectra of GO and two representative GO-IL composites (i.e., GO-[AEMIM][Br] and GO-[APMIM][NTf(2)]). As shown in Fig. 2(a), the strong peak at approximately 3500 cm^−1^ refers to the stretching vibration of the –OH group on the GO surface. The peak at 1729.97 cm^−1^ belongs to the C-O stretching vibration of the carboxyl group and that at 1623.14 cm^−1^ is attributed to the C=C bonds in the aromatic moieties^38^,^39^. In Fig. 2(b) and (c), the peak at 1167.11 cm^−1^ is associated with the ring in-plane asymmetric stretching of the imidazolium ring^40^. The relatively wide peak at 1617.68 cm^−1^ and the peak at 1652.15 cm^−1^ can be assigned to –CONH–37. These results suggest that the ILs have been successfully grafted onto the GO surface.

Figure S1 shows the Raman spectra of GO and two GO-IL composites in which a laser excitation of 532 nm was used. In the three samples, two strong peaks at approximately 1595 cm^−1^ and 1353 cm^−1^ are clearly visible, which are caused by the G and D bands, respectively. Relative to GO, a red-shift in the D-band of the GO-IL composites is observed. This phenomenon can be partly attributed to the linkage of the C and N atoms, which changes the electronic structure of GO. The intensity ratios of the two peaks (*I*_D_/*I*_G_) reflect the extent of defects on the GO surface and express the degree of covalent binding. The *I*_D_/*I*_G_ ratio of GO and the GO-IL composites are 1.01, 1.05 and 1.04, respectively, corresponding to a slightly increased value. Therefore, the increased ratios of *I*_D_/*I*_G_ show an increasing disorder and indicate the successful formation of GO-IL composites via the amidation reaction.

**Figure 3:**
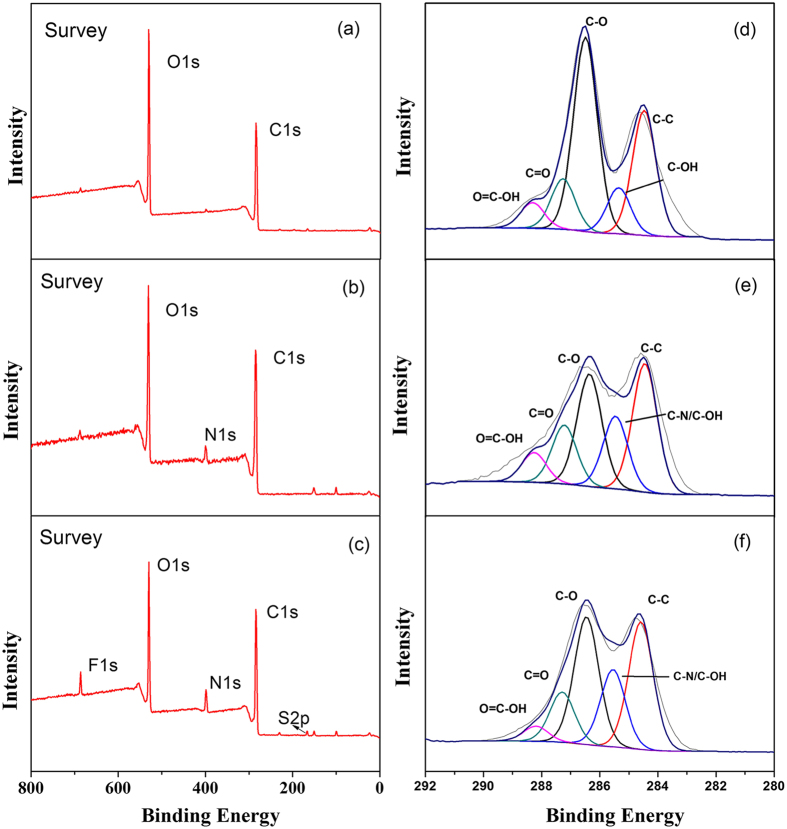
XPS spectra of (**a**) GO, (**b**) GO-[AEMIM][Br], and (**c**) GO-[APMIM][NTf(2)]; C1s XPS spectra of (**d**) GO, (e) GO-[AEMIM][Br], and (**f** ) GO-[APMIM][NTf(2)].

GO and the GO-IL composites were investigated by XPS. We note that only the C1s and O1s peaks exist in GO (Fig. 3a). The C1s peaks are obtained by fitting the five peaks at 284.8, 285.5 286.8, 287.8, and 288.7 eV, which are assigned to C-C (non-oxygenated ring C), C-OH, C-O, C=O, and O=C-OH^41^, as shown in Fig. 3d. The N1s peak can be observed in GO-[AEMIM][Br] (Fig. 3b). Relative to GO, the peak fitting of C1s in GO-[AEMIM][Br] yields a new functional group (i.e., C-N, Fig. 3e.), which is attributed to the successful amidation reaction between [AEMIM][Br] and GO. The N content increases dramatically from 0.68% to 5.10% whereas the O content decreases, as shown in Table S1. Similar results are obtained with GO-[APMIM][NTf(2)].

**Figure 4:**
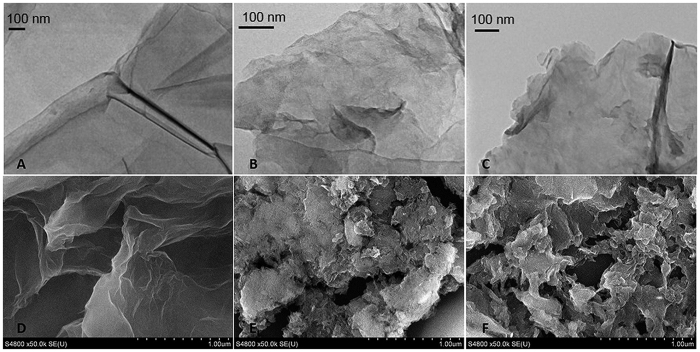
TEM images of (**A**) GO, (**B**) GO-[AEMIM][Br], and (**C**) GO-[APMIM][NTf(2)]; SEM images of (**D**) GO, (**E**) GO-[AEMIM][Br], and (**F**) GO-[APMIM][NTf(2)].

As shown in Fig. 4A and D, the TEM and SEM images indicate that the prepared GO possesses a semitransparent flake-like shape with a wrinkled single-layer structure. After being grafted by the ILs, the GO-[AEMIM][Br] and GO-[APMIM][NTf(2)] composites still maintain the lamellar structure shown in Fig. 4B and C, which ensures that they retain a high specific surface area and high adsorptive performance. A clear change in the SEM images can be observed. Fig. 4E and F show that the large GO sheets are reduced to small pieces, giving the appearance of different sized holes. This phenomenon can explain the low pressure in the SPE process. The characterizations demonstrate that these novel composites can be utilized as SPE adsorbents by overcoming the issue of high pressure, while retaining the advantage of the high adsorptive capacity of GO.

N_2_-soption isotherms and the corresponding pore size distribution curves are illustrated in Fig. S2. Relative to GO, the BET specific surface areas of GO-[AEMIM][Br] were found to be up significantly. The reason for this is that the binding to ionic liquids enhances the distance between the layer to layer of GO^40^. From Fig. S3, we can clearly see the 2 theta degree of the diffraction peak of GO-IL less than that of GO. This also confirms an increasing interlayer spacing of GO-IL in contrast to GO. GO and GO-[AEMIM][Br] demonstrate the type-IV isotherms with a typical hysteresis loop. The increased area of hysteresis loop of GO-[AEMIM][Br] indicates the larger pore size. And Fig. S2 (B) also proves that the pore size of GO transfer to bigger section after reaction with [AEMIM][Br]. As a result, there are great contributions in the adsorption capacity.

### Comparison of the Four GO-ILs Composites for the SPE

**Figure 5:**
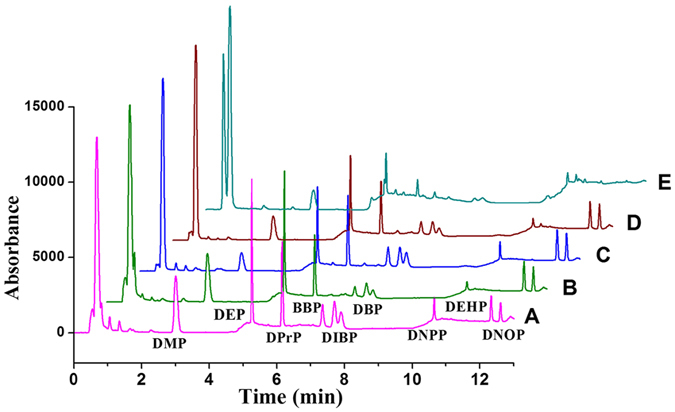
Chromatograms of the extracts of nine PAE standard aqueous solutions in 100 ng/mL by four GO-ILs composites and magnetic-GO: (**A**) GO-[AEMIM][Br], (**B**) GO-[AEMIM][NTf(2)], (**C**) GO-[APMIM][Br], (**D**) GO-[APMIM][NTf(2)], (E) magnetic-GO.

Ionic liquids are composed of cation and anion. So the optimal choice of ionic liquids for PAEs extraction rely on the design of the cation and anion. Functional amine ionic liquids were primarily chosed to bond with carboxy group on the surface of graphene oxide. And the large imidazolium ring with different alkyl chains as the cation can offer high π-π and hydrogen-bond interactions and enhance electrostatic inter-sheet repulsion which mean an increased adsorption capacity. As shown in Fig. 5, we find that the two [Br] anion ionic liquids possess a superior extraction capacity, especially for DMP, DEP, DPrP, BBP, DIBP, DBP and DNPP (Fig. 5). The extraction capacity of GO-[AEMIM][Br] is higher. The results indicate that a decreased carbon chain length benefits the extraction. The primary influence to the adsorptive performance of the GO-IL composites was the nature of the anion. The hydrophobic effect restrained the extraction process in the aqueous system^42^, As for the extraction of DNP and DNOP, the four GO-based composites had high capacities. Magnetic-GO (MGO) material (Fig. S4) was also prepared to aim at a comparison of GO-ILs and MGO’s extraction performances. We found that MGO’s adsorption abilities for these nine PAEs are significantly weaker than the four GO-ILs’, especially for BBP, DEHP and DNOP. For these reasons, we selected GO-[AEMIM][Br] as the SPE adsorbent.

### Optimization of SPE

**Figure 6:**
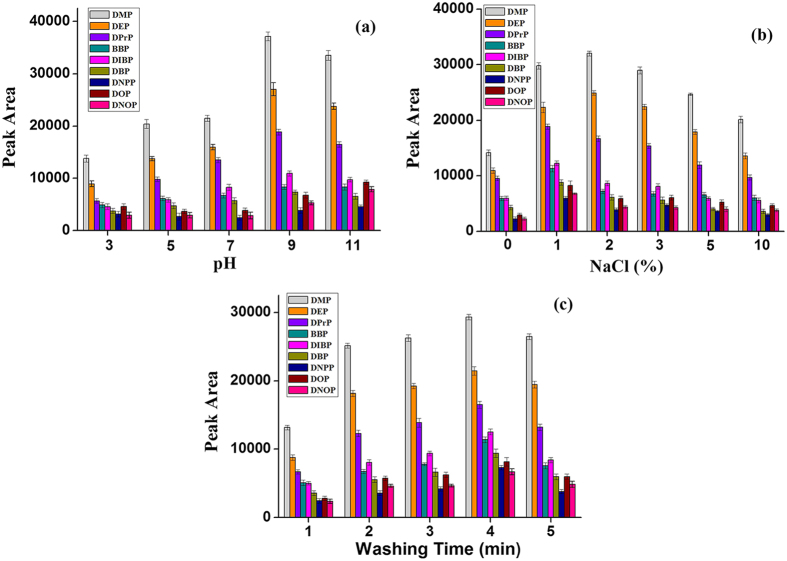
Optimization of pH (**a**), salt concentration (**b**), and washing time (**c**) for the extraction efficiency of nine PAEs.

Solid Phase Extraction is a dynamic partitioning equilibrium of the analytes in the aqueous phase and the solid phase. The common influential parameters such as pH values, salt concentration and washing time were investigated to acquire the optimal extraction performance of the GO-[AEMIM][Br] for the extraction of nine PAEs. In this work, the pH values of the sample solutions were evaluated in the range of 3.0–11. As shown in Fig. 6a, the peak areas of the PAEs after extraction clearly increased as the pH increased from 3.0 to 9.0. However, most of the peak areas decreased as the pH became greater than 9.0. As previously reported^43^, PAEs are relatively independent of changes to the sample solution in the pH range of 2.0–10, in which they exist as neutral molecules. Consequently, the pH value primarily influences the charge of the ionic liquids and other functional groups such as hydroxyl, carboxyl and epoxide groups on the GO surface. For this reason, a pH of 9.0 was adopted for the adsorption. The salt concentration is a significant factor that influences an extraction procedure. As shown in Fig. 6b, with 1% (w/v) sodium chloride (NaCl) added to the sample solution, the extraction performance of most of the target PAEs reached a maximum. This result occurs because of the salt-out effect, which often promotes extraction. However, when the NaCl concentration exceeds 2% (w/v), the mass transfer process in the solid/liquid interface becomes inhibited from the increased viscosity, leading to a reduction of the diffusion rate of the target PAEs, which decreases the extraction efficiency^44^. Therefore, 1% (w/v) NaCl was added to the sample solution. Figure 6c shows the effect of washing time on the extraction performance by changing the elution time from 1 min to 5 min. On the basis of the experimental results, the elution time was set to 4 min to ensure an optimal balance between time and efficiency.

### Reusability of GO-[AEMIM][Br]

**Figure 7:**
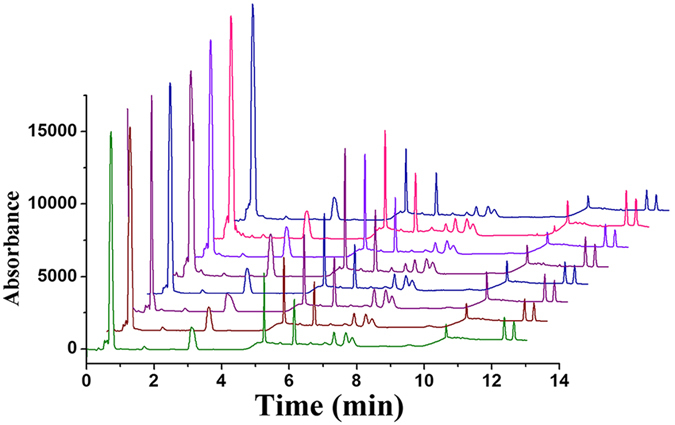
Chromatograms of the PAE extracts by recycling [AEMIM][Br]-GO eight times.

Reusability is a necessary parameter for estimating the extraction performance of GO-[AEMIM][Br]. According to Fig. 7, the adsorbent was used effectively 8 times for the extraction of nine PAEs (prior to the next use, the adsorbent was washed with 1 mL of methanol and 1 mL of water). The results remained nearly identical without an apparent loss of the adsorption capacity, except for a slight fluctuation for DNPP. In addition, as shown in Table S2, the adsorption capacities for nine PAEs by GO-[AEMIM][Br] were evaluated. Different concentrations of PAE standard solutions were processed by the method. The maximum adsorption ranged from 266.1 μg g^−1^ to 483.6 μg g^−1^. This demonstrates the excellent reusability and desirable adsorption capacity of the adsorbent.

### Validation of the Method

The analytical parameters of the SPE-HPLC-UV method for the determination of the PAEs, such as linearity, correlation coefficients (r^2^), limits of detection (LODs), limits of quantitation (LOQs) and repeatability were performed under the optimal experimental conditions by using a series of spiked water samples. As shown in Table 1, the linearity of the nine PAEs ranged from 2 ng mL^−1^ to 200 ng mL^−1^, with the correlation coefficients exceeding 0.9932. The LODs and LOQs were defined as the corresponding concentration equivalent to three and ten times the signal-to-noise ratios, which ranged from 0.02 ng mL^−1^ to 0.88 ng mL^−1^ and from 0.06 ng mL^−1^ to 2.94 ng mL^−1^, respectively. The sensitivity of the method, which uses a UV detector, is quite satisfying, especially because the detector is easily available to most analytical laboratories. The reproducibility of the method was determined by intra-day RSDs (n = 6) and inter-day RSDs (n = 6) at a spiked sample concentration of 50 ng mL^−1^. The two RSD values were always less than 8.2%. All of the results indicated a high sensitivity and good reproducibility of the method.

### Real Sample Analysis

**Figure 8:**
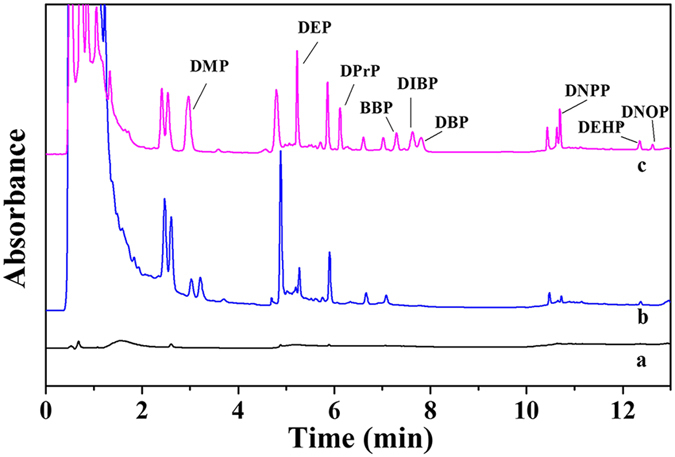
The chromatograms for the eraser 1 sample solution. (**a**) Injection without SPE; (**b**) Injection after SPE; (**c**) Spiked with 50 ng mL^−1^ of PAEs.

After validation, the method was applied to detect the PAE contents in three erasers. The erasers were purchased from a local supermarket and pretreated as previously described. As shown in Table 2, the eraser 1 solution contained 11.4 ng mL^−1^ of DEP and 16.9 ng mL^−1^ of DEHP. The calculated values of DEP and DEHP in eraser 1 were 11.4 mg kg^−1^ and 16.9 mg kg^−1^, respectively. The values of the spiked recoveries for the PAE analyses in the erasers ranged from 95.0% to 107.1%, with RSDs (n = 4) from 4.1% to 7.8%, which ensured the reliability of the proposed method. Eraser 2 contained 58.8 mg kg^−1^ of DMP, 31.1 mg kg^−1^ of DEP, 13.6 mg kg^−1^ of BBP and the concentration of DNPP exceeded the linear range. Eraser 3 contained 18.0 mg kg^−1^ of DMP, 12.9 mg kg^−1^ of DEP and 23.0 mg kg^−1^ of DEHP. Notably, the selected sample possessed a PAE content less than the regulatory limit of 0.1%. The representative chromatograms of eraser 1 before and after SPE and the spiked sample are shown in Fig. 8. To evaluate the present method, Table 3 shows a comparison with other procedures reported in the literature for the determination of PAEs. The improved sensitivity and linear ranges of the present method indicate its ease and superiority. Meanwhile, an obvious advantage is that this method only utilizes an HPLC/UV instrument, which is easily available to most laboratories.

## Conclusions

In conclusion, a series of ionic liquid modified graphene oxide composites were prepared through a direct amidation reaction. For the first time, the prepared composites were used as the adsorbent in a fixed-bed column, which possessed the advantages of low column pressure and high adsorption capacity. The system was successfully applied to the extraction of PAEs from eraser samples with good reproducibility, wide linear range and low LODs using a standard HPLC-UV detector. The proposed method provides a reliable method for the removal and determination of PAEs in aqueous solution, which can be applied in water treatment and the regulation of supplies. Moreover, this research highlights the use of novel types of GO-based materials for use in SPE.

## Methods

### Materials and Reagents

Graphite powder, KMnO_4_, NaNO_3_, H_2_SO_4_, HCl, DMF, N,N-dicyclohexylcarbodiimide (DCC), N-ethyl-N-(3-(dimethylamino) propyl) carbodiimide (EDC) and N-hydroxysuccinimide (NHS) were purchased from Sinopharm Chemical Reagent Co. Ltd. (Shanghai, China). The ionic liquids 1-aminopropyl-3-methylimidazolium bromide [APMIM][Br], 1-aminoethyl-3-methylimidazolium bromide [AEMIM][Br], 1-aminopropyl-3-imidazolium bis(trifluoromethylsulfonyl)imine [APMIM][NTf(2)] and 1-aminoethyl-3-methylimidazolium bis(trifluoromethylsulfonyl)imide [AEMIM][NTf(2)] were purchased from the Lanzhou Institute of Chemical Physics (Lanzhou, China). The standard PAE samples dimethyl phthalate (DMP), diethyl phthalate (DEP), dipropyl phthalate (DPrP), dibutyl phthalate (DBP), diisobutyl phthalate (DIBP), di(2-ethylhexyl) phthalate (DEHP), di(n-pentyl) phthalate (DNPP), di(n-octyl) phthalate (DNOP) and benzylbutyl phthalate (BBP) were purchased from ANPEL Laboratory Technologies Incorporation (Shanghai, China). Chromatographic grade acetonitrile, methanol and isopropanol were purchased from Sigma-Aldrich (St. Louis, MO). All the aqueous solutions were prepared using ultrapure water (18.2 MΩ/cm).

### Synthesis of ILs-modified GO composites

GO was prepared following a modified Hummers method^13^. Briefly, four types of ILs were modified on GO through an amidation reaction between the amino groups of the ILs and the carboxyl groups of GO. The two ILs associating with the bromide anion were hydrophilic. Thus, the two GO-IL composites were synthesized in the aqueous-phase and EDC/NHS were used as the coupling agent. This approach closely follows a previously reported work with some modification. First, 25 mg of GO was dispersed in 50 mL of water by ultrasonication for 2 h. Then, 100 mg of EDC and 80 mg of NHS were added to the homogeneous solution. The solution was magnetically stirred for 1 h to activate the carboxyl groups of GO. Then, 100 mg of [APMIM][Br] or [AEMIM][Br] was introduced and the mixture was ultrasonicated for 20 min. The mixture was then stirred at 50 °C for 4 h. The final product was washed several times with deionized water and methanol.

The two ILs associating with the [NTf(2)]^−^ anion are hydrophobic. The amidation reaction was performed in the organic-phase and DCC was used as the coupling agent. A mass of 20 mg of GO was dispersed in 50 mL of DMF by ultrasonication for 2 h. Then, 20 mg of DCC and 100 mg of [APMIM][NTf(2)] or [AEMIM] [NTf(2)] were added to the solution, and the reaction was performed at 50 °C for 30 h. The final product was washed several times with DMF and methanol.

### Characterization and HPLC measurements

The morphology of GO and the GO-IL composites were characterized on a HITACHIS-4800 scanning electron microscope (SEM, Hitachi Co. Ltd., Tokyo, Japan), a JEM-2010 transmission electron microscope (TEM, JEOL Ltd., Japan). FT-IR spectra were obtained on a Thermo Nicolet iS50 Fourier-transform (FT) infrared spectrometer (Madison, WI, USA). Raman spectra were recorded on a Nicolet 6700/NXR FT-Raman spectrometer (Thermo Electron, USA) with a laser excitation of 532 nm. XPS measurements were recorded on an AXIS Ultra DLD (Shimadzu-Kratos, Japan) with an Al athode as the X-ray excitation source.

Powder X-ray diffraction patterns (XRD) analysis were performed on a D8 advance diffractometer (Bruker AXS, Germany) at the voltage of 40 kV. Specific surface areas were calculated by the Brunauer–Emmett–Teller (BET) method using a Quadrasorb evo instrument (Quantachrome, USA) and the pore size distributions were determined from the related adsorption isotherms by using the Barrett–Joyner–Halenda (BJH) model.

HPLC analyses were performed using ultra-fast liquid chromatography (LC-30A, Shimadzu, Japan) with an auto sampler and a UV detector using a deuterium lamp as the light source (228 nm). The separation of nine PAEs was conducted on a Shimadzu shim-pack XR-ODS III (1.6 μm) column. Data processing was performed on a Lenovo computer running the LabSolutions software developed by Shimadzu. The separation conditions were optimized using a binary mobile phase composed of ultrapure water (18.2 MΩ/cm, solvent A) and acetonitrile (solvent B). The LC gradient elution program was as follows: 0–3.7 min = 35% B, 3.7–4 min = 35–70% B, 4–6.5 min = 70% B, 6.5–7 min = 70–60% B, 7–9 min = 60% B, 9–9.5 min = 60–100% B, 9.5–15 min = 100% B. The injection volume was 5 μL, and the flow rate was 0.3 mL/min.

### SPE Procedure and Real Sample Preparation

After preparation, 10.0 mg of the GO-IL composite was ground and packed into a standard filter, which acted as a homemade SPE column. The column was preconditioned with 1 mL of methanol and 1 mL of water. Then, 5 mL of the sample solution was perfused through the column at a flow rate of 1 mL/min. The adsorbed PAEs were eluted by 0.5 mL of methanol and the obtained extract was concentrated to dryness under a steam of nitrogen before detection. Glass materials (instead of plastic) were used for the entire procedure to avoid possible interferences.

Three erasers from different manufacturers were purchased from a local supermarket. The erasers were cut into small pieces and 0.1 g of the sample was placed into a glass flask with 100 mL of pure water. The mixtures were stirred at 37 °C for 72 h to stimulate the contact conditions an eraser in use would experience. The supernatant was removed and the pH value was adjusted to 9.0. An appropriate amount of NaCl was also added. Finally, the solution was processed as described above.

## Additional Information

**How to cite this article**: Zhou, X. *et al*. Ionic liquids modified graphene oxide composites: a high efficient adsorbent for phthalates from aqueous solution. *Sci. Rep.* **6**, 38417; doi: 10.1038/srep38417 (2016).

**Publisher's note:** Springer Nature remains neutral with regard to jurisdictional claims in published maps and institutional affiliations.

## Supplementary Material

Supplementary Material

## Figures and Tables

**Figure 1:**
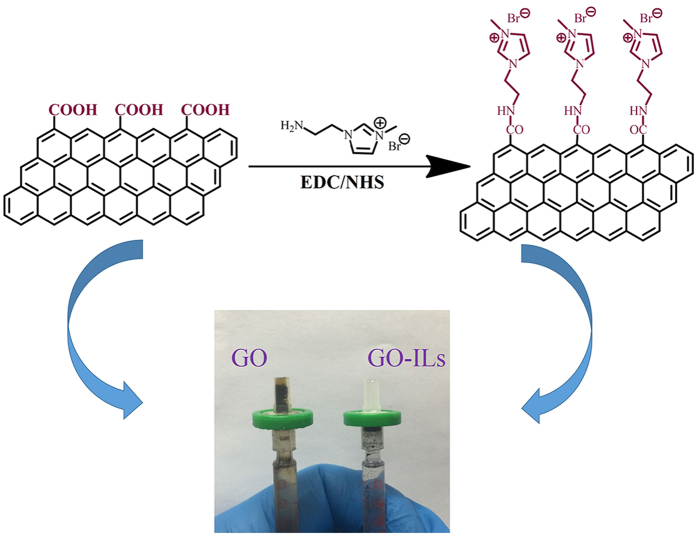
Schematic diagram of the preparation of GO-IL SPE column. Graphene oxide were reacted with [AEMIM][Br] to synthesize the GO-[AEMIM][Br] composite. And GO and GO-[AEMIM][Br] were packed into a syringe filter (0.45 μm, hydrophobic polytetrafluoroethylene membrane) respectively. In contrast to GO-ILs columns, GO column is easily broken during perfusion which is attributed to the high column pressure.

**Table 1:** Analytical parameters of the SPE-HPLC-UV method for the determination of nine PAEs in spiked water.

Analytes	Linear range (ng/mL)	Calibration equations	r^2^	LODs (ng/mL)	LOQ (ng/mL)	Intra-day RSDs (%)	Inter-day RSDs (%)
DMP	5–100	y = 773 ± 29x + 21151	0.9959	0.02	0.06	5.4	6.6
DEP	2–100	y = 753 ± 18x + 1011	0.9976	0.03	0.09	3.9	5.8
DPrP	2–200	y = 436 ± 6x−27	0.9989	0.05	0.16	3.7	6
BBP	5–200	y = 244 ± 7x−1188	0.9967	0.1	0.32	6.5	7.5
DIBP	5–200	y = 266 ± 7x−48	0.9971	0.08	0.26	5.1	7.2
DBP	5–200	y = 182 ± 6x + 231	0.9957	0.11	0.37	6.2	6.4
DNPP	10–200	y = 46 ± 2x + 2080	0.9948	0.24	0.8	6.6	7.1
DEHP	10–200	y = 49 ± 2x − 24	0.9932	0.43	1.44	4.8	5.7
DNOP	10–200	y = 25 ± 1x − 57	0.9944	0.88	2.94	8	8.2

**Table 2:** Determination of nine PAEs in real samples.

	eraser 1 (ng/mL)	RSD (%)	Spiked 20 ng/mL		RSD (%)	Spiked 50 ng/mL		RSD (%)	eraser 2 (ng/mL)	RSD (%)	eraser 3 (ng/mL)	RSD (%)
			found (ng/mL)	recovery (%)		found (ng/mL)	recovery (%)					
DMP			21.2	106.0	5.4	51.3	102.6	5.4	58.8	3.4	18.0	4.8
DEP	11.4	6.1	31.8	101.4	5.6	58.6	95.5	5.0	31.1	4.8	12.9	7.4
DPrP			21.0	105.0	4.8	49.4	98.8	6.4				
BBP			19.3	96.2	4.1	51.3	102.6	5.7	13.6	6.2		
DIBP			19.3	96.5	6.8	49.0	98.0	7.6				
DBP			20.2	100.8	7.7	53.4	106.7	6.5				
DNPP			21.4	107.1	5.6	52.8	105.6	6.5	>200			
DEHP	16.9	5.7	35.3	95.7	6.7	63.6	95.1	5.8			23.0	4.3
DNOP			19.0	95.0	7.8	47.7	95.4	6.1				

**Table 3:** Comparison of the current method with other reported methods for screening of phthalates.

Extraction Methods	Matrix	Detection	Linear ranges (ng mL^−1^)	LODs (ng mL^−1^)	RSDs (%)	Ref. (%)
Mag-Fe3O4@mSiO2-C18 SPE	Water	HPLC/UV	50–3000	25–77	4.9–11.2	[45]
Magnetic graphene SPE	Water	GC/MS	—	—	—	[46]
Poly(EGDMAMATrp) SPE	Saliva	GC/MS	100–2000	3–10	1.2–6.9	[47]
Waters Oasis MAX SPE cartridge	Serum	GC/MS	5–1000	0.7–4.5	4.9–13.3	[48]
Magnetic dummy molecularly imprinted microspheres dSPE	Beverages	GC/FID	4–400	0.74–1.2	3.1–6.9	[49]
Magnetic carbon nanotubes SPE	Urine	GC/MS	0.250–250	0.025–0.050	6.16–11.24	[50]
GO-ILs SPE	Water	HPLC/UV	2–200	0.02–0.88	3.7–8.2	Present
