# Supplementary material for: Ionic liquids modified graphene oxide composites: a high efficient adsorbent for phthalates from aqueous solution
Source: Sci Rep. 2016 Dec 2;6:38417. doi: 10.1038/srep38417 (PMC5133574; doi:10.1038/srep38417)
Supplement: Supplementary Material [file srep38417-s1.pdf]

Ionic liquids modified graphene oxide composites: a high efficient adsorbent for phthalates from aqueous solution

Xinguang Zhou, Yinglu Zhang, Zuteng Huang, Dingkun Lu, Anwei Zhu, and Guoyue Shi*

School of Chemistry and Molecular Engineering, East China Normal University, 500 Dongchuan Road, Shanghai 200241, P.R. China

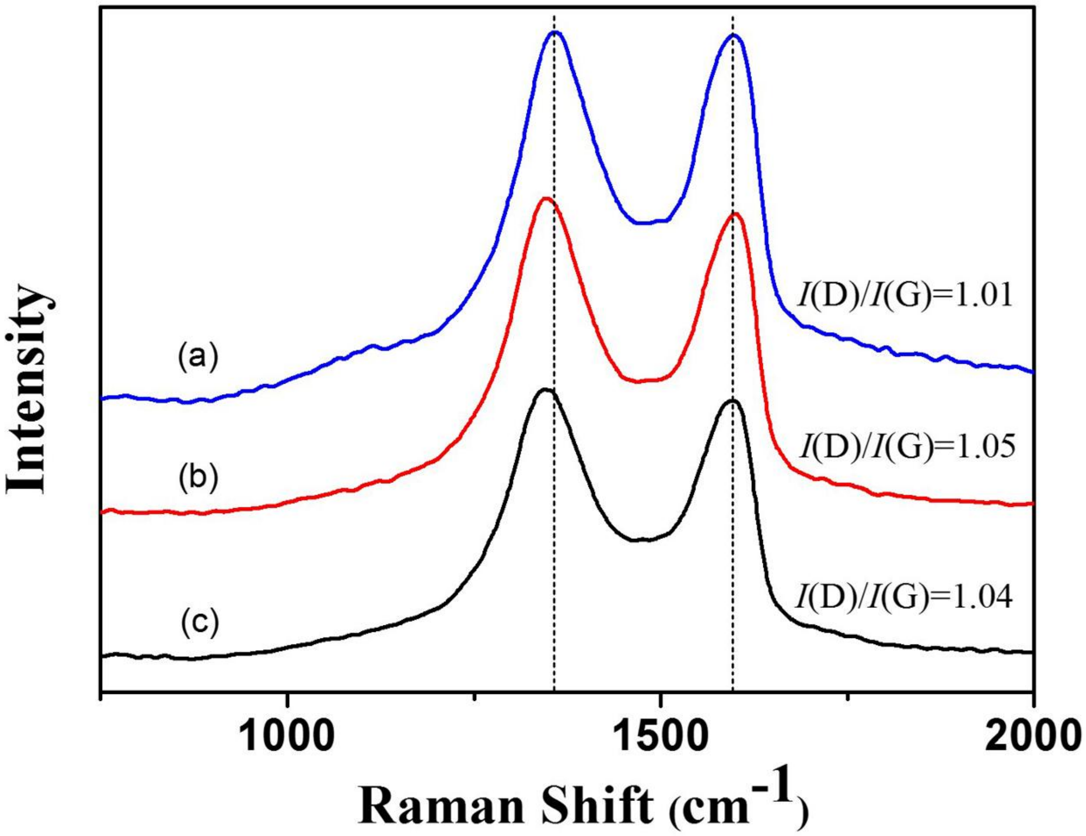

Figure S1. Raman spectra of (a) GO, (b) GO-[AEMIM][Br], and (c) GO-[APMIM][NTf(2)].

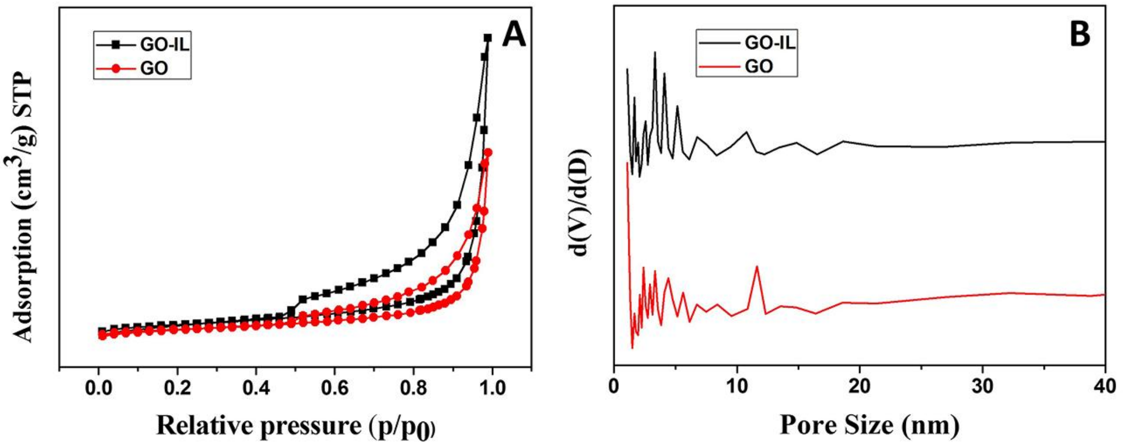

Figure S2. (A)N2-sorption isotherms of GO and GO-[AEMIM][Br] (B) the corresponding pore size distribution curves.

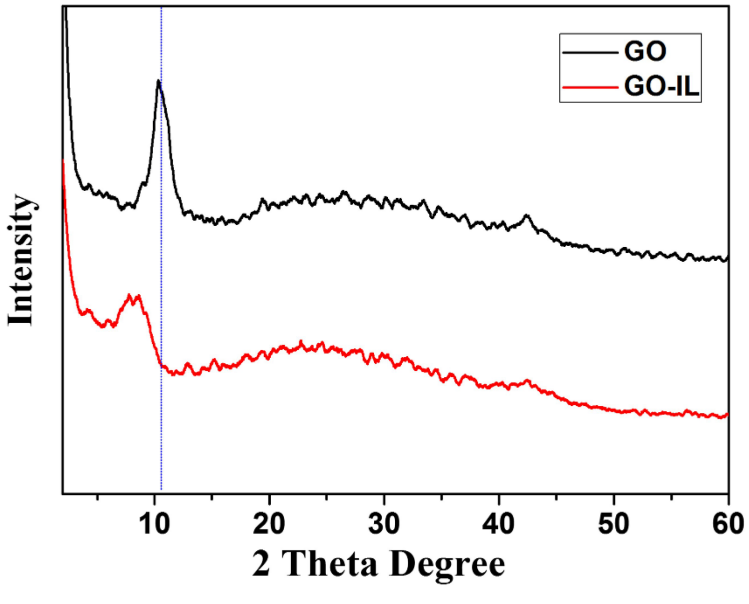

Figure S3. XRD patterns of the GO and GO-[AEMIM][Br].

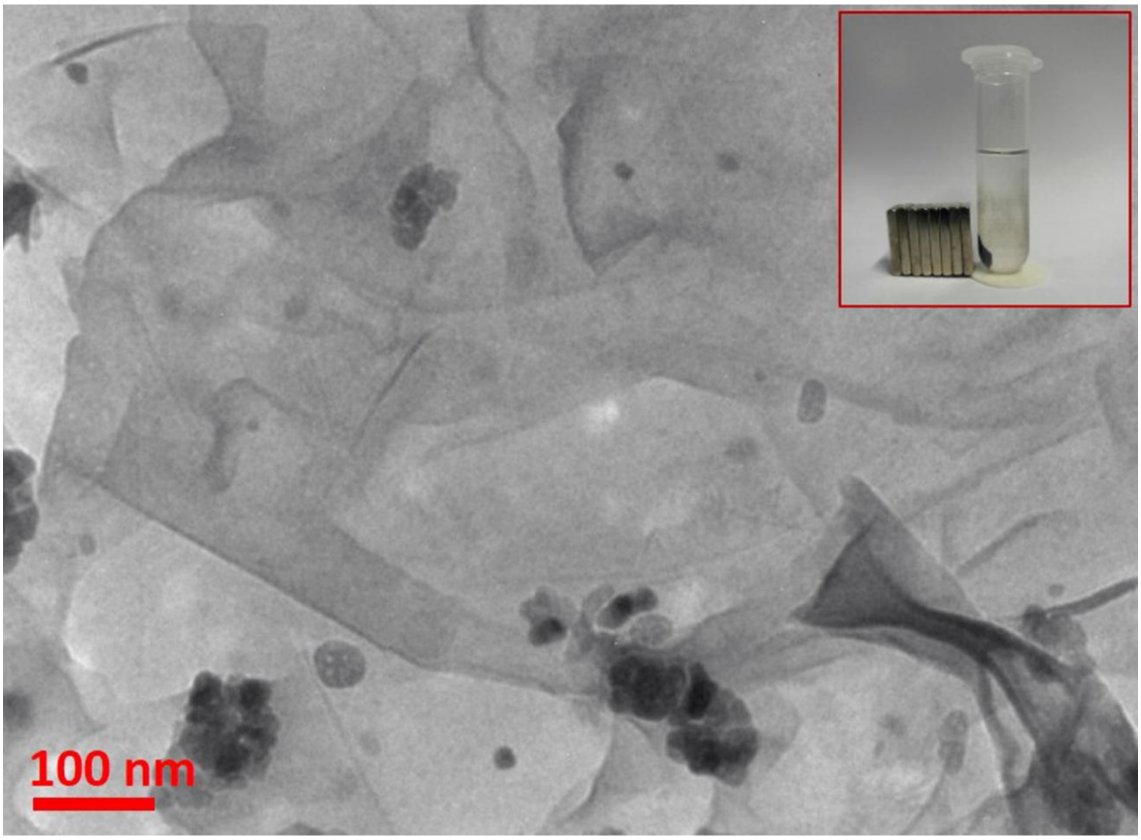

Figure S4. TEM image of MGO.

Table S1. The content of element C, O, N in GO and GO-ILs composites.

	C (%)	O (%)	N (%)
GO	70.75	28.56	0.68
GO-[AEMIM][Br]	72.00	21.35	6.65
GO-[APMIM][NTf(2)]	72.24	21.26	5.10

Table S2. The adsorption capacities for nine PAEs by GO-[AEMIM][Br]

	1 μg/mL	2 μg/mL	4 μg/mL	6 μg/mL	10 μg/mL
	μg/g	μg/g	μg/g	μg/g	μg/g
DMP	42.9	141.1	182.4	262.4	273.1
DEP	45.5	124.2	160.8	267.0	336.1
DPrP	68.6	169.6	220.4	346.4	404.9
BBP	92.0	180.0	317.6	470.1	459.7
DIBP	84.3	172.6	268.4	374.8	342.5
DBP	84.6	162.8	254.2	385.3	368.9
DNPP	84.5	169.2	333.7	483.6	448.2
DDP	68.2	137.0	266.1	207.9	101.5
DNoP	81.4	144.2	277.9	215.0	112.0
